# *Mycobacterium tuberculosis* Induces *Irg1* in Murine Macrophages by a Pathway Involving Both TLR-2 and STING/IFNAR Signaling and Requiring Bacterial Phagocytosis

**DOI:** 10.3389/fcimb.2022.862582

**Published:** 2022-05-02

**Authors:** Caio C. B. Bomfim, Logan Fisher, Eduardo P. Amaral, Lara Mittereder, Katelyn McCann, André A. S. Correa, Sivaranjani Namasivayam, Muthulekha Swamydas, Mahtab Moayeri, Jonathan M. Weiss, Raj Chari, Daniel W. McVicar, Diego L. Costa, Maria R. D’Império Lima, Alan Sher

**Affiliations:** ^1^Department of Immunology, Institute of Biomedical Sciences, University of São Paulo, São Paulo, Brazil; ^2^Laboratory of Parasitic Diseases - National Institute of Allergy and Infectious Diseases, National Institutes of Health, Bethesda, MD, United States; ^3^Laboratory of Clinical Immunology and Microbiology - National Institute of Allergy and Infectious Diseases, National Institutes of Health, Bethesda, MD, United States; ^4^Department of Biochemistry and Immunology - Ribeirão Preto Medical School, University of São Paulo, São Paulo, Brazil; ^5^Graduate Program in Basic and Applied Immunology - Ribeirão Preto Medical School, University of São Paulo, São Paulo, Brazil; ^6^Laboratory of Cancer Immunometabolism, Center for Cancer Research, National Cancer Institute, Frederick, MD, United States; ^7^Laboratory Animal Sciences Program, Frederick National Laboratory for Cancer Research, Frederick, MD, United States

**Keywords:** Irg1, *Mycobacterium tuberculosis*, macrophages, TLR-2, ESAT-6, STING, IFN, ESX-1 system

## Abstract

Irg1 is an enzyme that generates itaconate, a metabolite that plays a key role in the regulation of inflammatory responses. Previous studies have implicated Irg1 as an important mediator in preventing excessive inflammation and tissue damage in *Mycobacterium tuberculosis* (*Mtb*) infection. Here, we investigated the pattern recognition receptors and signaling pathways by which *Mtb* triggers Irg1 gene expression by comparing the responses of control and genetically deficient BMDMs. Using this approach, we demonstrated partial roles for TLR-2 (but not TLR-4 or -9), MyD88 and NFκB signaling in Irg1 induction by *Mtb* bacilli. In addition, drug inhibition studies revealed major requirements for phagocytosis and endosomal acidification in Irg1 expression triggered by *Mtb* but not LPS or PAM3CSK4. Importantly, the *Mtb*-induced Irg1 response was highly dependent on the presence of the bacterial ESX-1 secretion system, as well as host STING and Type I IFN receptor (IFNAR) signaling with Type II IFN (IFN-γ) signaling playing only a minimal role. Based on these findings we hypothesize that *Mtb* induces Irg1 expression in macrophages *via* the combination of two independent triggers both dependent on bacterial phagocytosis: 1) a major signal stimulated by phagocytized *Mtb* products released by an ESX-1-dependent mechanism into the cytosol where they activate the STING pathway leading to Type I-IFN production, and 2) a secondary TLR-2, MyD88 and NFκB dependent signal that enhances Irg1 production independently of Type I IFN induction.

## Introduction

Tuberculosis (TB) is an airborne infectious disease caused by *Mycobacterium tuberculosis* (*Mtb*) that despite the existence of prophylactic and therapeutic measures remains a serious public health problem. Although an estimated quarter of the global population is infected with *Mtb*, most individuals remain asymptomatic with only 5-10% developing active TB in their lifetimes (WHO, 2020). This has suggested that the host immune response is sufficient to contain bacterial infection in most cases and that its modulation may explain disease progression. Indeed, it is well known that the balance between pro-inflammatory and anti-inflammatory responses is crucial in determining the outcome of *Mtb* infection. Weak activation of the immune response such as that occurring in acquired immunodeficiency syndrome (AIDS) patients and individuals treated with immunosuppressive drugs promotes rapid bacterial proliferation and dissemination (Keane et al., 2001; Bell and Noursadeghi, 2018; Goletti et al., 2018). On the other hand, an excessive inflammatory response may trigger necrotic issue damage which also can promote bacillary spread (Orme et al., 2015).

Previous studies have showed that itaconate plays a major role in the regulation of macrophage-dependent inflammation (Lampropoulou et al., 2016; Bambouskova et al., 2018; Dominguez-Andres et al., 2019). The production of this metabolite is determined by immune responsive gene 1 (*Irg1*) that encodes an enzyme highly expressed in response to pro-inflammatory stimuli and converts the tricarboxylic acid (TCA) cycle intermediate cis-aconitate into itaconate. Recent studies have demonstrated a protective role for Irg1 in the response to *Mtb* in that mice deficient in the *Irg1* gene display increased susceptibility and lowered survival (Nair et al., 2018; Hoffmann et al., 2019). In addition, Irg1 expression and itaconate production by LysM^+^ myeloid cells reduce the lung immunopathology mediated by excessive neutrophil recruitment during murine infection (Nair et al., 2018).

In addition to its immunomodulatory activity, itaconate also has been reported to display bacteriostatic effects on *Mtb* when cultivated in liquid medium where acetate is the only source of carbon (Michelucci et al., 2013). This was hypothesized to result from itaconate inhibition of isocitrate lyase (ICL) (Williams et al., 1971; Rittenhouse and McFadden, 1974; McFadden and Purohit, 1977), an enzyme expressed by some prokaryotic microorganisms that is essential for their survival in glucose deficient environments such as the phagosome. ICL shifts bacterial metabolism from glycolysis to the glyoxylate pathway in order to obtain energy from β-oxidation of fatty acids or acetate in the absence of other carbon sources (Hillier and Charnetzky, 1981). Indeed, *Mtb* bacilli express high levels of ICL after uptake by macrophages and cannot grow in the absence of this enzyme in both *in vitro* and *in vivo* conditions (McKinney et al., 2000; Hou et al., 2002; Munoz-Elias and McKinney, 2005). Consistent with this hypothesis, Hoffmann and colleagues have demonstrated that *Mtb* replication is increased in Irg1-deficient macrophages and dendritic cells (Hoffmann et al., 2019).

Despite the considerable evidence for an important role of Irg1-itaconate axis in host resistance to *Mtb*, the molecular signals that drive Irg1 expression in macrophages, the major host cell infected by this pathogen are not completely understood. Under non-infectious conditions, Irg1 is upregulated in classically activated M1 macrophages and itaconate production inhibits M2 macrophage polarization (Ganta et al., 2017; Puchalska et al., 2018; Runtsch and O’Neill, 2019). Further studies in human cells (monocytes, macrophages and dendritic cells) demonstrated that a wide variety of proinflammatory signals which drive M1 macrophage polarization, such as toll-like receptor (TLR) agonists [lipopolysaccharide (LPS) and poly-IC] as well as some pro-inflammatory cytokines [Tumor Necrosis Factor Alpha (TNF-α), interferon-beta (IFN-β) and interferon-gama (IFN-γ)], can promote Irg1 expression (Tallam et al., 2016; Mills et al., 2018; Salyer and David, 2018). In particular, Irg1 is known to be strongly induced by IFNs, and studies in human peripheral blood mononuclear cell (PBMC)-derived macrophages stimulated with LPS identified IFN regulatory factor 1 (IRF1) as an important transcriptional regulator of Irg1 expression (Tallam et al., 2016). In addition, a synergistic interaction between TLR and IFN signaling has been reported (Tallam et al., 2016; Mills et al., 2018). This view of Irg1 induction has been further corroborated in murine macrophages genetically deficient in different innate signaling molecules. The latter studies have implicated both MyD88 and TRIF as well as the type I IFNAR receptor as important elements for Irg1 induction in LPS-stimulated macrophages (Kawai et al., 2001; Hirotani et al., 2005; Mills et al., 2018). In the case of *Mtb* infection, previous studies have shown that the pathogen stimulates strong Irg1 expression in murine macrophages (Shi et al., 2005; Nair et al., 2018; Hoffmann et al., 2019). Studying the response of *Mtb*-infected bone marrow-derived macrophages (BMDMs), Shi and colleagues reported that Irg1 induction by *Mtb* is dependent on signaling by IFNAR, but not by TLRs or other pattern recognition receptors they tested (Shi et al., 2005).

In the present study, we have re-examined in greater detail the process whereby *Mtb* triggers Irg1 expression in murine macrophages. Our findings reveal that the stimulation of Irg1 response by the pathogen occurs through the combined action of two signaling pathways both dependent on bacterial phagocytosis. The first involves the ESAT-6-mediated release of phagocytozed *Mtb* products into the cytosol, and their sensing by the stimulator of interferon genes (STING) pathway that triggers Type I IFN production. The second involves a previously unappreciated contribution of TLR2-mediated MyD88-NFκB-dependent signaling to the Irg1 response.

## Materials and Methods

### Mice

C57BL/6J, Thy1.1 C57BL/6J, NFkBp50^-/-^, IFNAR^-/-^ male mice were provided by the NIAID Taconic Farms Animal supply Contract (Hudson, NY, USA). TLR-2^-/-^, TLR-4^-/-^ and TLR-9^-/-^ mice were kindly provided by Dr. Giorgio Trinchieri and CARD9^-/-^, Sting^-/-^ and IFNγR^-/-^ mice by Drs. Michail Lionakis, Mahtab Moayeri and Dragana Jankovic (NIAID), respectively. Irg1-GFP mice were developed by CRISPR/Cas9 mediated generation of an Acod1-C-terminal GFP fusion mouse. The genomic sequence corresponding to the Acod1 locus (Accession number: NM_008392.1) was downloaded from the UCSC Genome Browser. The sequence surrounding the C-terminal exon was used to identify putative guide RNA sequences using sgRNA Scorer 2.0 (PMID: 28146356). Candidate sequences were identified and tested in P19 cultured cells for editing efficiency. Two candidate sequences (protospacer adjacent motif in bold); (CACGGTGGAAAGCCTTATAACGG) and (GAAACAGAGACAAGCGTATATGG) were used for mouse model generation. A long ssDNA donor containing approximately 500 bp of homology on each side of the stop codon, linker, GFP sequence and silent mutations in the sgRNA target sites was created using the Guide-it production kit (Clontech), which was complexed to Cas9 protein. Cas9 protein was expressed from the SP-Cas9 expression plasmid (Addgene #62731) (PMID: 25910214) by the Protein Expression Laboratory at the Frederick National Lab for Cancer Research. SP-Cas9 was a gift from Niels Geijsen (Addgene plasmid # 62731; http://n2t.net/addgene:62731; RRID:Addgene_62731). Embryos were injected with the ssDNA:Cas9 complex and surgically transferred to recipient mice within 24 hr. Tail biopsies from the recipient mice were used for PCR genotyping using these primers: Left forward (5’-AACAAACAGATCTTGGACCTG-3’), Left reverse (5’-CGTCGTCCTTGAAGAAGATG-3’), Right forward (5’-CATCTTCTTCAAGGACGACG-3’), Right reverse (5’-GAAGACATTCAGCGTGGTTG-3’).

All mice were on a C57BL6 genetic background and bred and housed in NIH animal biosafety level (ABSL) 2 or 3 facilities and used at 8-11-weeks old. Male mice were employed in all experiments. All studies were conducted in accordance with protocol LPD-99E approved by the NIAID Animal Care and Use Committee. All animals were maintained under specific pathogen-free conditions with *ad libitum* access to water and food. C57BL/6J or Thy1.1 C57BL/6J mice were used interchangeably in different experiments with indistinguishable results.

### Bacterial Culture

The H37Rv strain of *Mtb* and BCG (Pasteur strain) were grown in Middlebrook 7H9 plus Tween 80 (0.05%) enriched with 10% ADC (albumin, dextrose and catalase). The genetically modified fluorescent reporter strain H37Rv-RFP was originally provided by Dr. Joel Ernst (University of California, USA) and grown in Middlebrook 7H9 enriched with 0,05% tween 80, 10% ADC and 30 µg/ml kanamycin (Sigma-Aldrich, USA). All bacterial strains were maintained in culture at 37°C until the midlog phase (OD 0.6-0.9) on day 7.

### BMDM Culture

BMDMs were prepared according to previously published protocols (Amaral et al., 2019). Briefly, bone marrow cells were harvested from femurs and tibia of mice by flushing with 1X PBS. A single-cell preparation was obtained by careful cycling through a 26-gauge needle. Cells were then cultivated in petri dishes (100 x 15 mm) with DMEM/F-12 (Gibco, USA) supplemented with sodium pyruvate (Gibco, USA; 1 mM), L-glutamine (Gibco, USA; 2 mM), 1% Hepes (Life Technologies, USA), gentamicin (Gibco, USA; 25μg/ml) and 10% fetal bovine serum (FBS) and 20% L929 supernatant media and incubated at 37°C and 5% CO_2_. L929 cell-conditioned medium (10 ml) was added on day 4 and the cells were cultured for another 3 days to achieve their full differentiation into macrophages.

### *In Vitro* Infection and Treatments

BMDMs were detached by flushing with cold PBS and seeded in 24-well plates at 10^6^ cells/well. In some experiments, cells were treated with phagocytosis inhibitors [1 µM mycalolide B (Enzo Life Sciences, USA) (Tosh et al., 2016); or 40 µM dynasore (Sigma Aldrich, USA)] for 1 h prior to bacterial infection, or with inhibitors of gene transcription [1 μg/ml actinomycin D (Sigma Aldrich, USA)] or protein synthesis [10 μg/ml cycloheximide (Sigma Aldrich, USA)] for 2 h before infection. In other experiments BMDMs were treated with recombinant IFN-β (R&D Systems – USA) or IFN-γ (R&D Systems – USA) concomitant with infection. BMDMs were infected with H37Rv, H37RV-RFP or BCG strains routinely at a MOI of 1:1 for 3 h, washed and then cultivated for additional 3 h, or stimulated with LPS (10 ng/mL). In some experiments, *Mtb* was opsonized with fresh or heat-inactivated (30 min, 56°C) C57BL/6 mouse sera for 1 h prior to macrophage exposure.

### Preparation of Human Macrophages

Human elutriated monocytes were obtained from peripheral blood of healthy donors from the NIH blood bank under a protocol approved by NIAID and the Department of Transfusion Medicine, and were differentiated into macrophages as previously described (Amaral et al., 2019). Briefly, CD14^+^ monocytes were purified by magnetic column, plated in 96-well plates (Corning, USA) containing RPMI 1640 medium (Life Technologies, USA) and supplemented with 10% human AB serum (Corning) and human rM-CSF (50 ng/ml; Peprotech, USA) for 7 days with addition of fresh media with the indicated growth factors every 48 h. Differentiated macrophages were then infected with H37Rv bacilli (routinely at MOI=1:1).

### *In Vivo* Mtb Infection

C57BL/6 (BL/6) mice were infected *via* aerosol with approximately 100 H37Rv bacilli, in a whole body inhalation system (Glas Col, USA). Mouse infection was confirmed by plating lung homogenates obtained at day 0 of infection on 7H11 agar medium (Sigma-Aldrich) enriched with 0.5% glycerol (Mallinckrodt Pharmaceuticals, USA) and 10% OADC (BD Biosciences, USA) media.

### Measurement of Gene Expression by RT-PCR

Total RNA was extracted from cells or lung homogenates using RNA isolation kits (Zymo Research, USA) and RT-PCR performed as previously described (Mayer-Barber et al., 2014). Briefly, the cells or the post-caval lobe of mice lung were lysed in 300 µl of TRIZOL (Life Technologies) and passed through columns (Zymo Research, USA) according to the manufacturer’s protocol. Total RNA was extracted by disrupting the pulmonary tissue in 2 ml tubes containing 2.7 mm glass bead and TRIZOL, using RNA isolation kit. The isolated RNA was quantified and diluted to 100 ng/µl. For cDNA conversion, dNTP mixture, oligo (dT), random primers and superscript II reverse transcriptase (Thermo Fisher Scientific, USA) were used. Real time PCR reaction was performed on an 790HT Fast Real Time PCR device with Quant-Studio 7 Real-Time PCR Systems (Thermo Fisher Scientific, USA). *Irg1*, *Tnf*, *Il12p35*, *Ifnb1*, *Oas1a* and *Mx2* gene expression were determined by Sybr Green qRT-PCR (Applied Biosystems, USA), according to the manufacturer’s instructions. GAPDH was used as housekeeping gene and changes in mRNA expression between control and infected macrophages were calculated using the 2^^-ΔΔCT^ method (Fold change). The sequences of the specific primers employed are listed in **Table 1**.

**Table 1:** Sequences of PCR primers employed.

Gene	Forward Primer (5’- 3’)	Reverse Primer (5’- 3’)
*Gapdh*	TGAAGCAGGCATCTGAGGG	CTCCCACTCTTCCACCTTCG
*Tnf*	CATCTTCTCAAAATTCGAGTGACAA	GGGTTGTACCTTGTCTACTCCCA
*Ifng*	TCAAGTGGCATAGATGTGGAAGAA	CATGAAAATCCTGCAGAGCCA
*Irg1*	GCGAACGCTGCCACTCA	ATCCCAGGCTTGGAAGGTC
*Il12p35*	ACGTCTTTGATGATGACCCTGT	TTCTGAAGTGCTGCGTTGA
*Ifnb1*	GTCCGAGCAGAGATCTTCAGG	ACTACCAGTCCCAGAGTCCG
*Mx2*	CCAGTTCCTCTCAGTCCCAAGATT	TACTGGATGAAGGGAACGTGG
*Oas1a*	CCCTATCTGACACATTGACGGT	TATTCTATGGTCCCCCAGCCT

### Flow Cytometric Analysis

BMDMs (10^6^) were stained using fluorochrome-labeled monoclonal antibodies to CD11b and TLR2. In some experiments, cells were infected with H37Rv bacteria expressing red fluorescent protein (RFP). The samples were acquired using a FACS Fortesa flow cytometer (BD Biosciences, USA) and analyzed by FlowJo software (FlowJo LLC, USA).

### Statistical Analyses

Student’s *t* tests and One-Way ANOVA followed by Tukey’s post-test were performed using Prism 7 software (GraphPad Software Incorporated, USA). Data are shown as mean ± SEM values. Differences between groups were considered significant when the p-value was <0.05, with asterisks denoting the degree of significance (*P < 0.05; **P < 0.01 and ***P < 0.001).

## Results

### *Mtb* Is a Potent Inducer of Irg1 Expression in Murine and Human Macrophages *In Vitro* and in Infected Mice

Before dissecting the signaling pathways involved in Irg1 expression, we confirmed the ability of *Mtb* (H37Rv strain) to induce this response *in vitro* and *vivo*. High levels of *Irg1* gene message were observed in the lungs of *Mtb*-infected mice beginning at 3 weeks after aerosol infection (**Figure S1A**). Consistent with previous findings (Shi et al., 2005; Nair et al., 2018; Hoffmann et al., 2019), *Mtb* also induced potent *Irg1* gene expression in both human monocyte-derived macrophages (**Figure S1B**) and mouse BMDMs (**Figure S1C**). In the case of the murine cells, high *Irg1* transcript levels were observed as early as 3 h, peaking at 6-9 h and declining at 12 h after bacterial exposure. *Mtb* also induced *Tnf* and *Il12* gene expression with similar kinetics in the same macrophages, but the highest response of these cytokine messages proceeded the peak of *Irg1* expression by approximately 3h (**Figures S1D, E**). Remarkably, significant *Irg1* induction was observed at multiplicities of infection as low as 0.1:1 illustrating the potency of *Mtb* as a trigger of this gene (**Figure S1F**).

**Figure 1:**
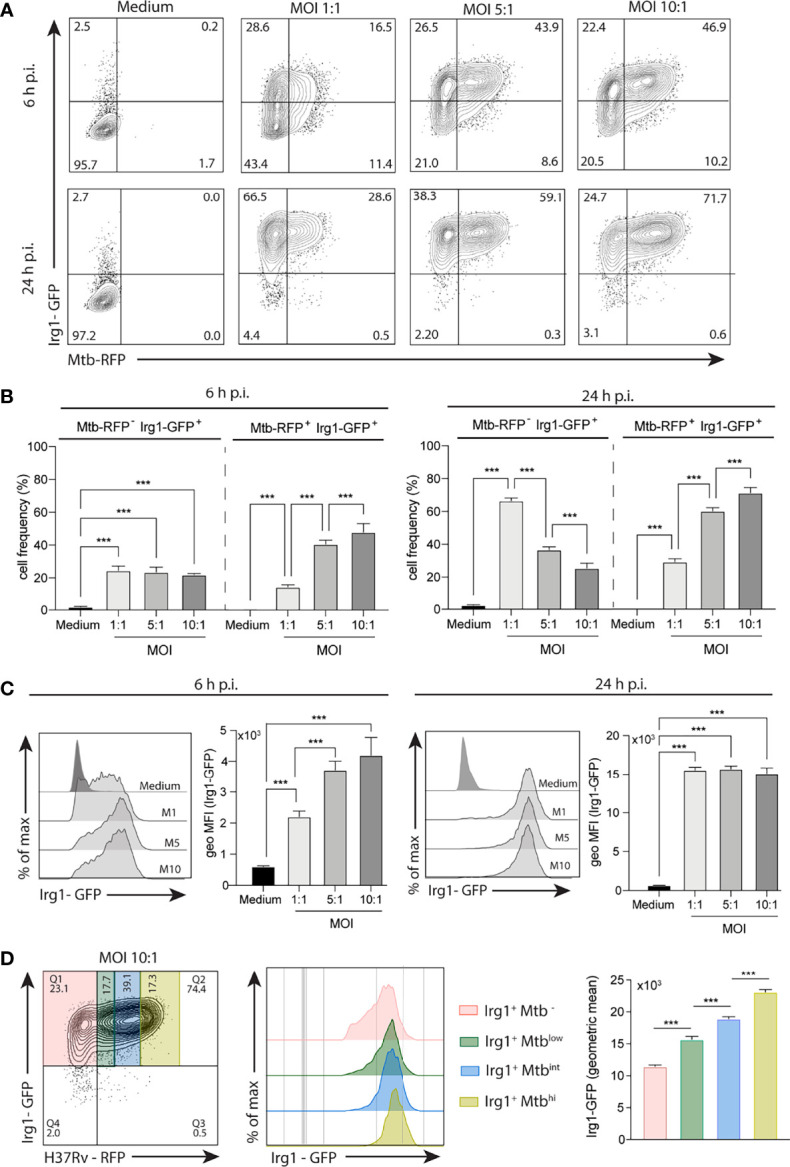
*Mtb* induces Irg1 protein expression in macrophages. BMDMs obtained from transgenic mice expressing Irg1-GFP protein (Irg1-GFP^+^) were infected with H37Rv-RFP at different MOI (1:1; 5:1; 10:1) as indicated. **(A, B)** Mtb-induced Irg1-GFP expression was evaluated by flow cytometry at 6 h and 24 h p.i. **(A)** Sample FACS plots and **(B)** frequency of macrophages expressing Irg1-GFP are shown. **(C)** Histogram FACS plot and geometric MFI of Irg1-GFP was evaluated by flow cytometry at 6 h (left) and 24 h (right) p.i. **(D)** FACS plot and histogram of Irg1-GFP expression in cells displaying low, intermediate and high MFI of RFP. Statistical significance was assessed by one-way ANOVA analysis for the indicated experimental condition (***p < 0.001). The data shown are from a representative experiment of two performed.

To demonstrate that *Mtb*-induced *Irg1* gene expression results in production of the Irg1 protein, we employed a recently developed GFP reporter mouse in which Irg1 production is associated with a fluorescent signal. BMDMs from these mice were exposed to H37Rv bacilli expressing RFP reporter. When examined at 6 h post-infection (p.i.), Irg1-GFP production by macrophages dramatically increased at MOIs as low as 1:1 and at 24 h most cells expressed the protein (**Figures 1A–C**). Interestingly, a sizeable fraction of Irg1-GFP^+^ BMDMs showed an uninfected *Mtb*-RFP^-^ phenotype, suggesting the existence of paracrine Irg1 induction (**Figures 1A, B**). Nevertheless, in infected BMDMs, the induced Irg1 protein levels directly correlated with the MFI of H37Rv-RFP expression (**Figure 1D**).

### *Mtb*-Induced *Irg1* Gene Expression in BMDMs Is Partially Dependent on TLR2-MyD88-NFκB Signaling

**Figure 2:**
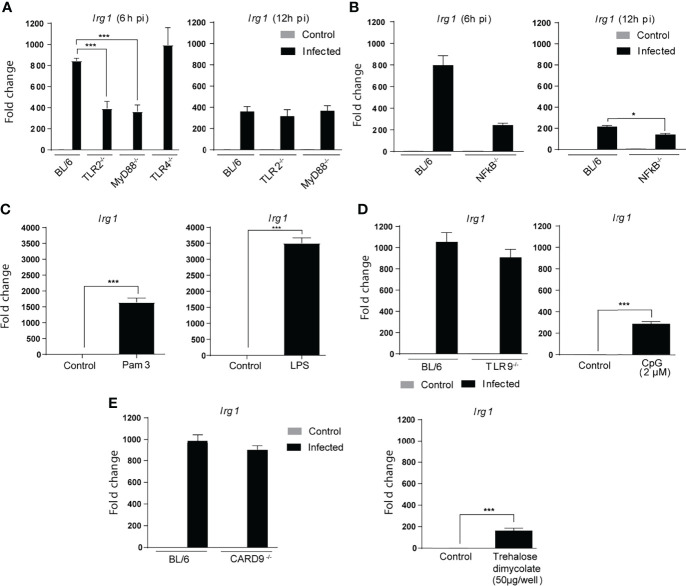
*Mtb* induced Irg1 expression is partially dependent on TLR-2/MyD88/NFκB signaling. **(A, B)** *Irg1* mRNA expression was assessed in C57BL/6, TLR-2^-/-^, MyD88^-/-^ and TLR-4^-/-^ and NFkB-p50^-/-^ BMDM cultures following H37Rv infection at 6 h and 12 h p.i. **(C)** Irg1mRNA levels were evaluated in C57BL/6 BMDMs at 6 h following Pam3CSK4 (10 ng/ml) or LPS (10 ng/ml) stimulation. **(D, E)** Irg1 mRNA expression was assessed in BMDMs from **(D)** C57BL/6, TLR-9^-/-^ and **(E)** CARD9^-/-^ mice infected with H37Rv strain (MOI of 1) at 6 h p.i. C57BL/6 BMDMs were stimulated with **(D)** CpG (2 µM) or **(E)** trehalose dimycolate (50 µg/well) and Irg1 expression evaluated at 6 h after stimulation. As a control both CpG and trehalose dimycolate were shown to induce significant TNF-α responses when tested under the same conditions (data not shown). Significant differences are indicated as follows: *p<0.05, and ***p < 0.001. Results are representative of at least two separate experiments performed.

The cellular response to *Mtb* has been shown to depend on recognition of the bacillus by pattern recognition receptors (PRR) located in the plasma membrane, endosomes and host cell cytoplasm (Means et al., 1999; Underhill et al., 1999; Schoenen et al., 2010; Collins et al., 2015; Basu et al., 2018; Wagener et al., 2018; Chai et al., 2020). The Toll-like receptor family plays a major role in this process triggering host gene expression largely through MyD88 dependent NFκB mediated signaling (Underhill et al., 1999; Scanga et al., 2004; Stamm et al., 2015). To assess the involvement of TLR signaling in *Mtb*-induced Irg1 expression, we compared BMDMs from MyD88 or NFκBp50-deficient mice with BL/6 macrophages. We observed at 6 h p.i. a partial but significant decrease in *Irg1* gene expression in the absence of MyD88 and NFκBp50 (**Figures 2A, B**). Analysis of the macrophage response at 12 h p.i. showed that this reduction in *Irg1* levels was not due to a delay in gene expression.

A major MyD88 dependent PRR triggered by *Mtb* is TLR-2 (Yu et al., 2014; Stamm et al., 2015) and consistent with its well documented importance (Underhill et al., 1999), BMDMs from TLR-2^-/-^ mice, but not from TLR-4^-/-^ or TLR-9^-/-^ mice, displayed a defective *Irg1* response to *Mtb* similar to MyD88^-/-^ and NFκB-p50^-/-^ macrophages (**Figures 2A, B**). In control experiments we showed that BMDMs produce significant *Irg1* responses to LPS and (**Figure 2C**) CpG oligonucleotides (**Figure 2D**) confirming that the failure of TLR-4 and TLR-9 deficiency to inhibit *Mtb* induced *Irg1* is not due to a generalized defect in the function of these TLR under the conditions employed (**Figure 2D**). In addition to TLR, a second major PRR family involved in *Mtb* innate recognition are the c-type lectins (CLR), and in particular dectin 1 and mincle, that signal through the adaptor caspase recruitment domain-containing protein 9 (CARD9) (Wagener et al., 2018). Nevertheless, CARD 9*^-/-^* BMDMs exposed to mycobacteria expressed *Irg1* at levels similar to control BL/6 macrophages, demonstrating that these CARD9-dependent CLR pathways are not required for the *Mtb*-induced *Irg1* response (**Figure 2E**, left panel). In addition, trehalose dimycolate the major *Mtb* ligand for mincle stimulated only marginal Irg1 expression (**Figure 2E**, right panel). Together, these experiments revealed a substantial and highly specific contribution of the TLR2-MyD88-NFκB pathway for *Irg1* induction in *Mtb*-infected macrophages, presumably occurring during initial contact of the pathogen with the host cell surface and/or endosomal membranes.

### Phagocytosis of *Mtb* Is Required for the Induction of *Irg1* Gene Expression

**Figure 3:**
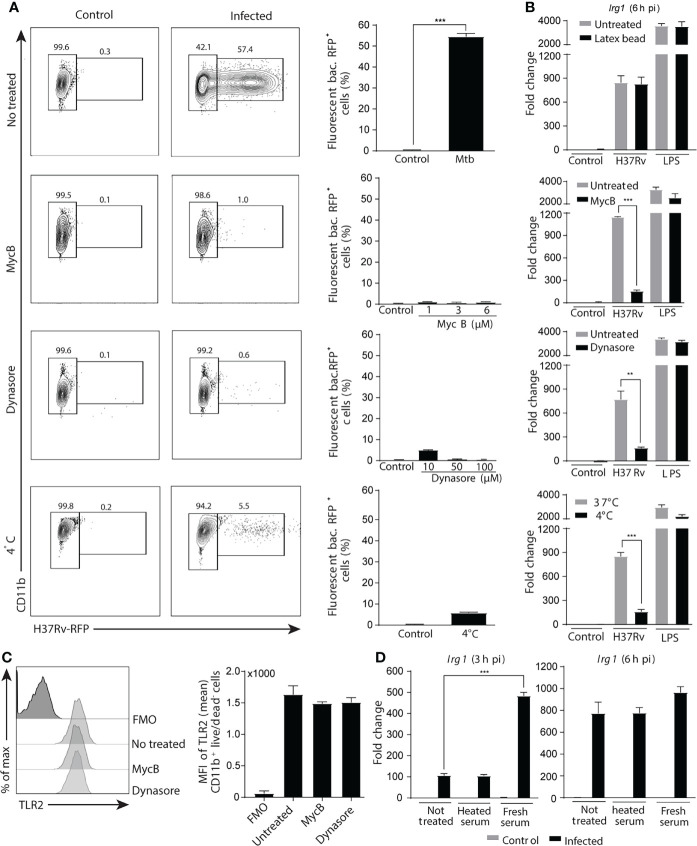
Phagocytosis of *Mtb* is crucial for the induction of Irg1 gene expression. **(A–C)** Macrophages were pretreated with the phagocytosis inhibitors Mycalolide B (MycB; 1 µM, 3 µM or 6 µM) or Dynasore (10 µM, 50 µM or 100 µM) for 1 h and then infected with H37Rv-RFP or stimulated with LPS (10 ng/ml) as indicated. **(A)** Phagocytosis was determined by flow cytometric analysis at 6 h after H37Rv-RFP Mtb exposure (MOI of 1). FACS plot (left panel) and summary data (right planel) are shown. **(B)** Irg1 expression was evaluated in BL/6 BMDMs exposed to latex beads (0.025%) (upper graph) or to H37Rv treated or not with MycB and Dynasore as well as in cultures left at 4 ^0^C. **(C)** TLR-2 protein expression by Mtb-infected macrophages following MycB and Dynasore treatment assessed by flow cytometry. **(D)** Irg1 mRNA levels were determined in BMDMs exposed to H37Rv opsonized or not with fresh or heat-inactivated naïve mouse sera at 3 h and 6 h p.i. Statistically significant differences are indicated as follows: **p < 0.01 and ***p < 0.001. Data are representative of two separate experiments.

*Mtb* infection of macrophages is initiated by phagocytosis of the bacteria that are able to survive by inhibiting phagosome-lysosome fusion and/or by escaping into the cytosol (Scherr et al., 2009; Zhai et al., 2019). Signaling by PRR present in endosomal membranes or in the cytoplasm thus could also be involved in stimulating Irg1 expression. To determine whether Irg1 induction requires phagocytic uptake of *Mtb*, BMDMs were treated with increasing concentrations of an actin polymerization inhibitor (mycalolide B) or a dynamin inhibitor (dynasore), or cells were maintained at 4°C before and during bacterial infection. All treatments markedly inhibited phagocytosis (**Figure 3A**), without effecting cell viability (**Figure S2A**). Importantly, phagocytosis inhibition was associated with a sharp decrease in *Irg1* gene expression induced by *Mtb* infection, whereas no change was observed for LPS-stimulated *Irg1* responses (**Figure 3B**). Moreover, phagocytosis of inert latex beads did not induce *Irg1* expression or enhance the response triggered by either *Mtb* or LPS (**Figure 3B**), demonstrating that phagocytosis in itself is not sufficient to trigger *Irg1*. Furthermore, phagocytosis inhibition did not affect cell surface TLR2 levels (**Figure 3C**), ruling out this possible explanation of the data. Finally, *Irg1* expression was increased by opsonization of *Mtb* with fresh mouse serum, but not with heat-inactivated serum (**Figure 3D**). The opsonization-enhanced *Irg1* response was seen at 3 h p.i. but not at 6 h p.i., suggesting that *Irg1* expression was accelerated by promoting mycobacterial phagocytosis through macrophage complement receptors. Indeed, phagocytosis assays performed with RFP-labelled *Mtb* showed that opsonization with fresh serum increased the frequency of infected macrophages in comparison with unopsonized bacteria or *Mtb* pre-incubated with heat inactivated serum when examined at 1h and 3h p.i. (**Supplementary Figure 2B**).

### STING-Dependent Cytosolic Sensing of *Mtb* Is a Second Signal Required for *Irg1* Gene Expression

**Figure 4:**
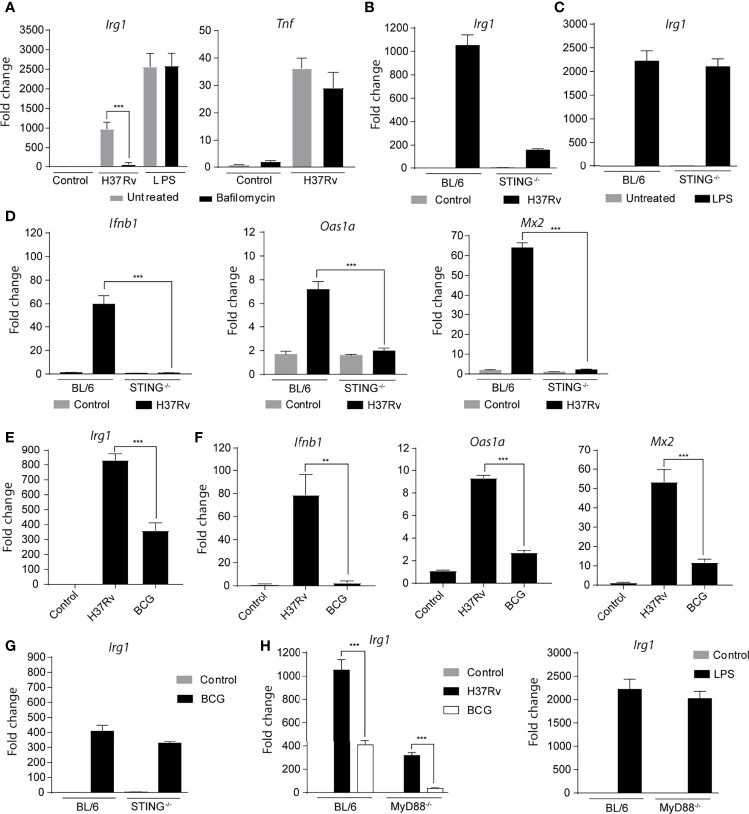
Cytosolic sensing of *Mtb* is a second major signal required for Irg1 gene expression. **(A)** Expression of *Irg1* and *Tnfa* at mRNA levels was evaluated in C57BL/6 BMDMs treated or not with bafilomycin (1 µM) and infected with H37Rv (MOI of 1) for 6 h. LPS stimulation (10 ng/ml) was used as a positive control for Irg1 and TNF-α induction in BMDM cultures. **(B**, **C)** *Irg1* gene expression in STING^-/-^ and C57BL/6 BMDMs at 6 h p.i. with H37Rv or stimulated with LPS (10 ng/ml). **(D)** *Ifnb1*, *Oas1a* and *Mx2* mRNA expression was assessed in STING^-/-^ and C57BL/6 BMDM cultures infected with H37Rv (MOI of 1) at 6 h p.i. **(E**, **F)** mRNA levels of *Irg1*, *Ifnb1*, *Oas1a* and *Mx2* were determined in C57BL/6 macrophages infected with H37Rv or BCG at 6 h p.i. **(G)** *Irg1* mRNA expression was measured in BCG-infected STING^-/-^ and C57BL/6 macrophage cultures at 6 h p.i. **(H)** Expression of *Irg1* was determined in MyD88^-/-^ and C57BL/6 BMDMs infected with H37Rv or BCG strains at MOI of 1, or stimulated with LPS (10 ng/ml) at 6 h p.i. or stimulation. Statistical significance was assessed by one-way ANOVA analysis for the indicated experimental condition (**p < 0.01 and ***p < 0.001). The data are representative of at least two separate experiments performed.

We next evaluated whether the presence of *Mtb* products in the cytosol is required to induce *Irg1* gene expression. First, we observed that the inhibition of phagosome acidification with bafilomycin reduced *Irg1* expression in *Mtb*-infected BMDMs, but not in LPS-stimulated BMDMs (**Figure 4A**). *Tnfa* gene expression was not affected by this treatment, revealing a preferential role for phagosome acidification in the induction of *Irg1* expression in *Mtb*-infected BMDMs (**Figure 4A**).

It is well established that *Mtb* from virulent strains, such as H37Rv, release molecules into the cytoplasm that activate cytosolic sensors by a mechanism dependent on the bacterial virulence factor ESAT-6 (Brodin et al., 2006; Groschel et al., 2016; Conrad et al., 2017). Therefore, we hypothesized that translocation of mycobacterial antigen from phagocytic vesicles into the cytosol and activation of cytosolic sensors could trigger *Irg1* expression in *Mtb*-infected macrophages. To investigate this possibility, *Irg1* responses were evaluated in *Mtb*-infected or LPS-treated BMDMs deficient in STING, a signaling adaptor activated by cGAS, a cytosolic DNA sensor stimulated during intracellular *Mtb* infection as a result of phagosomal permeabilization and required for its induction of Type IFN (Manzanillo et al., 2012; Collins et al., 2015; Watson et al., 2015; Cheng and Schorey, 2018). We observed markedly reduced levels of *Irg1* expression in *Mtb*-infected STING^-/-^ BMDMs compared to their control BL/6 counterparts (**Figure 4B**), indicating an important role for the STING activation pathway in this response. In contrast, the *Irg1* response to LPS was not altered in STING^-/-^ macrophages (**Figure 4C**). Also, in agreement with previous studies showing the requirement of STING for IFN-β production by *Mtb*-infected macrophages (Manzanillo et al., 2012; Collins et al., 2015; Watson et al., 2015), no increase in *Ifnb1* expression and, consequently, in interferon-stimulated genes (ISGs) *Oas1a* and *Mx2* were observed after *Mtb* infection of STING^-/-^ BMDMs (**Figure 4D**).

To confirm that cytosolic sensing of *Mtb* antigens is crucial to the *Irg1* response in infected BMDMs, we used BCG, an avirulent strain of *Mycobacterium bovis* (*Mbv*) that lacks the genes encoding the ESX-1 secretion system which includes ESAT-6 as a major component, and consequently, is unable to release antigens into the cytosol and activate STING. We observed that *Irg1* expression was lower in BCG-infected BL/6 BMDMs compared to H37Rv-infected BL/6 BMDMs (**Figure 4E**), as was the induction of *Ifnb1*, *Oas1a* and *Mx2* expression (**Figure 4F**). Furthermore, *Irg1* was expressed at comparable low levels in BCG-infected BL/6 and STING^-/-^ BMDMs, consistent with the inability of these mycobacteria to activate STING (**Figure 4G**). Interestingly, no induction of *Irg1* expression was observed in BCG-infected MyD88^-/-^ BMDMs (**Figure 4H**), perhaps because TLR2 and STING signaling are both absent in this situation. In contrast, BL/6 and MyD88^-/-^ BMDMs expressed high *Irg1* levels after LPS stimulation, suggesting the involvement of the alternative TRIF pathway of TLR4 signaling in the case of this agonist (**Figure 4H**).

### Type I IFN Signaling Induced by *Mtb* Infection Plays a Major Role in Enhancing *Irg1* Expression

**Figure 5:**
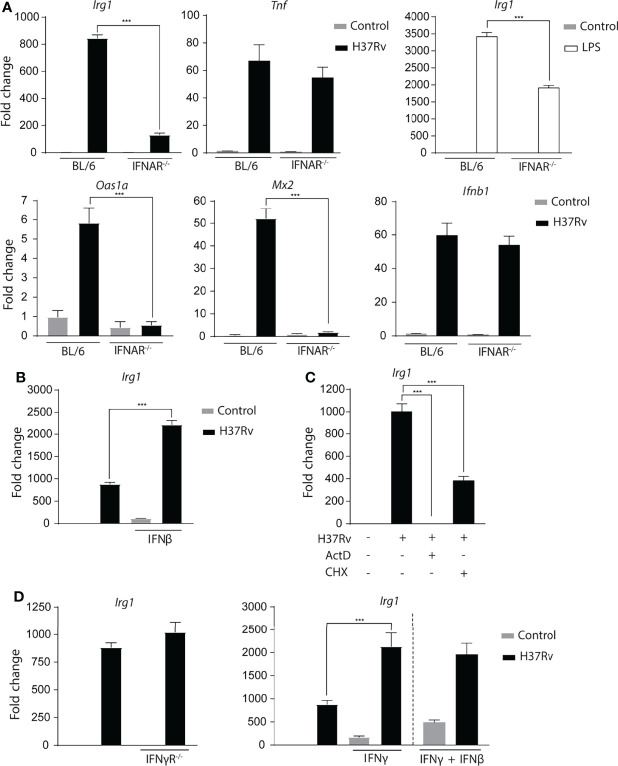
*Mtb*-induced Type I interferon signaling plays a major role in Irg1 expression. **(A)** mRNA levels of *Irg1*, *Tnf*, *Ifnb1*, *Oas1a* and *Mx2* were measured in IFNAR^-/-^ and C57BL/6 BMDMs at 6 h following H37Rv *Mtb* infection or LPS stimulation (10 ng/ml). **(B)** *Irg1* mRNA expression was assessed in C57BL/6 BMDMs treated or not with recombinant IFN-β (10 ng/ml). **(C)** mRNA levels of Irg1 were evaluated in C57BL/6 BMDMs pretreated with actinomycin D (ActD) (1 µg/ml) or cycloheximide (CHX) (10 µg/ml) for 2 h and then infected with H37Rv *Mtb* for 6 h. **(D)** *Irg1* mRNA expression was assessed in Mtb-infected IFNγR^-/-^ and C57BL/6 macrophage cultures in the presence of recombinant IFNγ (10U/ml) and/or IFNβ (10 ng/ml) at 6 h p.i. Significant differences are indicated with asterisks (***p < 0.001). The data shown are representative of two separate experiments performed.

As IFN-β responses are impaired in the absence of STING, BMDMs deficient in the type I interferon receptor (IFNAR^-/-^) were evaluated to determine whether this cytokine is involved in *Irg1* expression. In agreement with previously published observations (Shi et al., 2005), we observed that IFNAR^-/-^ BMDMs expressed substantially lower *Irg1* levels compared to BL/6 BMDMs after *Mtb* infection, whereas the *Tnf* response was not significantly affected. In addition, LPS-treated IFNAR^-/-^ BMDMs also displayed a diminished *Irg1* response (**Figure 5A**). This observation can be explained by the known ability of LPS to stimulate Type I IFNs *via* TRIF-induced triggering of IRF3 (Monroe et al., 2010; Hu et al., 2012). As expected, *Mtb*-infected IFNAR^-/-^ BMDMs also showed defective ISG (*Oas1a* and *Mx2*) expression along with unchanged *Ifnb1* levels (**Figure 5A**). Furthermore, stimulation with recombinant IFN-β substantially increased *Irg1* expression in *Mtb*-infected BL/6 BMDMs (**Figure 5B**) while at the concentration employed a minimal response was triggered in uninfected macrophages. Together these data reinforce and expand previous findings (Shi et al., 2005) indicating that Type I IFN signaling is an important regulator of *Irg1* expression during *Mtb* infection. Consistent with a *de novo* synthesis requirement of Type I IFNs for *Irg1* induction, addition of the protein synthesis inhibitor cycloheximide at the time of infection partially suppressed the Irg1 response to *Mtb*, which as expected was fully blocked by the transcription inhibitor actinomycin D (**Figure 5C**).

Because in addition to Type I IFN, Type II IFN is also a potent inducer of Irg1 in macrophages (Price et al., 2019), the response of *Mtb*-infected IFNγR^-/-^ BMDMs was also assessed. The results indicated that in contrast to Type I signalling, IFN-γ signalling does not contribute to the *Irg1* response to *Mtb* (**Figure 5D**). That Mtb induces insufficient amounts of IFN-γ to trigger autocrine activation of BMDMs was suggested by the observation that stimulation with the recombinant cytokine enhanced *Irg1* expression in both uninfected and *Mtb*-infected BMDMs (**Figure 5D**). Moreover, combined IFN-γ plus IFN-β treatment did not further potentiate *Irg1* expression over stimulation with IFN-γ alone. Together, these data indicate that *in vitro* Type I but not Type II IFN, play a major role in driving the Irg1 response in *Mtb*-infected macrophages, although it remains possible that *in vivo Irg1* expression can be be enhanced by paracrine IFN-γ produced by other cell types.

### *Mtb* Induces *Irg1* Expression by Triggering Combined But Mutually Independent TLR2 and STING Signaling Pathways

**Figure 6:**
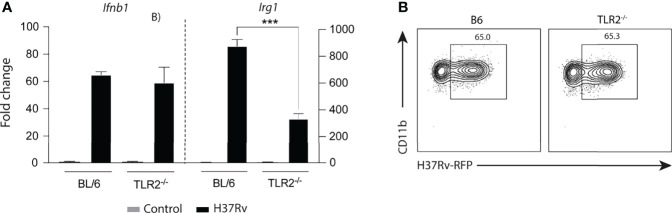
*Mtb* induced expression of *Ifnb1* as well as macrophage bacterial uptake are TLR-2 independent processes. **(A)** *Ifnb1* and *Irg1* mRNA levels were assessed in TLR-2^-/-^ and C57BL/6 BMDM cultures infected with H37Rv (MOI of 1) at 6 h p.i. **(B)** Internalization of H37Rv-RFP in TLR-2^-/-^ and C57BL/6 macrophages was measured by flow cytometry at 6 h p.i. Significant differences are indicated with asterisks (***p < 0.001). Results shown are representative of two independent experiments performed.

Our data indicated that two main phagocytosis-dependent signaling pathways involving the TLR-2/MyD88/NFκB and STING/IFN-I/IFNAR axes take part in the Irg1 response to *Mtb* infection. To investigate the interdependence of these signaling pathways, *Ifnb1* expression was assessed in *Mtb*-infected TLR-2^-/-^ and control BL/6 BMDMs. Because no difference in *Ifnb1* expression was observed between these two macrophage types (**Figure 6A**), we concluded that TLR-2 does not mediate its effects by triggering Type I IFN production in *Mtb*-infected BMDMs. Moreover, TLR2 signaling was not required for phagocytosis of *Mtb*, which was similar in TLR-2^-/-^ and BL/6 BMDMs (**Figure 6B**). Together, these findings demonstrate that TLR2 signaling by *Mtb* promotes *Irg1* expression in macrophages through a STING-independent signaling pathway.

## Discussion

Irg1 is heavily induced by *Mtb* infection where it is thought to play a key role in suppressing both neutrophilic inflammation and bacterial growth through its generation of itaconate (Nair et al., 2018; Hoffmann et al., 2019). As an IFN responsive gene, *Irg1* expression is enhanced in myeloid cells by Type I and Type II IFNs (Naujoks et al., 2016; Tallam et al., 2016; Mills et al., 2018). During the adaptive immune response to *Mtb* production of the enzyme is likely driven in large part by T cell derived IFN-γ. However, in the innate response to this pathogen Irg1 could be induced directly in myeloid cells or indirectly by IFNs acting in autocrine or in paracrine fashion on neighboring uninfected cells. In the present study we have examined in detail the pathways responsible for the innate induction of Irg1 by *Mtb* in murine BMDM. We demonstrate that *Mtb* triggers Irg1 expression directly through TLR2-/MyD88/NF-κB signaling and indirectly through STING dependent Type I IFN synthesis. While stimulation of both of these pathways depends on bacterial phagocytosis as evidenced by the near total ablation of the Irg1 response as a consequence of phagocytosis inhibition (**Figure 3**), the two pathways appear to be unlinked at least at the level of IFN induction. Their combined function may contribute to the particularly potent Irg1 response triggered by *Mtb* in macrophages.

Previous studies have documented the strong induction by *Mtb* of Irg1 mRNA expression in macrophages *in vitro* (Shi et al., 2003; Shi et al., 2005; Hoffmann et al., 2019). In the present report we both confirmed these findings and using macrophages from a newly developed reporter mouse extended them by demonstrating the co-ordinate induction of Irg1 protein expression (**Figure 1**). These experiments which employed fluorescent tagged *Mtb* revealed Irg1 synthesis in both infected and uninfected bystander macrophages in the culture suggesting the involvement of multiple autocrine and paracrine mechanisms for Irg1 stimulation.

In agreement with previous studies by Shi et al. (Shi et al., 2005) we observed that Type I but not Type II IFN signaling plays a major role in *Mtb* stimulation of Irg1 expression in murine macrophages. Exogenously added IFN-γ or IFN-β both augmented the Irg1 response suggesting that this response dichotomy may be due to the subthreshold induction of IFN-γ versus strong induction of Type I IFNs by *Mtb* in these host cells. Also, consistent with previous studies (Collins et al., 2015) we observed a major requirement for STING in *Mtb* induced IFN-β as well as Irg1 expression in our macrophage cultures implicating a critical role for the cGAS/STING/TBK-1/IRF-3 pathway of cytosolic DNA recognition (Marinho et al., 2017). Triggering of this pathway has previously been shown to depend on the bacterial virulence factor ESX-1, a secretion system which can mediate permeabilization of the phagosome membrane leading to the release of bacterial DNA into the cytosol. Indeed, BCG which lacks ESX-1 was defective in inducing both Irg1 and Type I IFN expression in macrophages and its residual stimulation of Irg-1 was not STING dependent.

Finally, consistent with the phagosome as the gateway into the cytosolic recognition pathway, Irg1 induction by *Mtb* was nearly totally eliminated when either phagocytosis or endosome acidification were blocked using chemical inhibitors while LPS induced Irg1 expression as expected was unaffected. Together these findings both confirm the major role for Type I IFN signaling in *Mtb* induced Irg1 expression described by Shi et al. while further demonstrating the critical involvement of phagocytosis and ESX-1 driven STING signaling in this pathway.

In addition to the role of cytosolic receptor recognition driven IFN signaling, we examined the contribution of membrane associated pattern receptors known to be stimulated by *Mtb* in macrophage expression of Irg1. Previous studies have argued against the involvement of both macrophage mannose and scavenger receptors (Shi et al., 2005) and the data presented here with CARD9 deficient mice provide additional evidence against a role for c-type lectin signaling in *Mtb* induced Irg1 expression. Moreover, trehalose dimycolate, a major agonist produced by *Mtb* failed to induce appreciable levels of Irg1 message in uninfected macrophages.

Previous work by Shi et al. had failed to detect a role for either MyD88, TLR-2 or TLR-4 in Irg1 induction in *Mtb* infected macrophages. In contrast we detected a partial but highly significant reduction in Irg1 expression in *Mtb* exposed MyD88, NF-κB and TLR-2 but not TLR4 or TLR-9 deficient macrophages. This response defect was evident at 6 but not 12 h post-infection. At present, the explanation for the discrepancy between our results and those of Shi et al. on the role of TLR signaling in Irg1 induction is unclear. Differences in the strain of *Mtb* (H37rv versus strain1254), routine MOI (1:1 vs 5:1) employed or differentiation status of macrophages are possible contributing factors that need to be systematically investigated to establish the generality of the role for TLR2 evidenced here. *Mtb* possesses potent TLR-2 ligands and in that sense the comparable phenotype of TLR-2 and MyD88 vs TLR-4 and TLR-9 deficient macrophages is not surprising. However, MyD88 independent pathways for TLR mediated IRG1 induction have also been described (Hoshino et al., 2002) and could have contributed to the effects we observed.

The observation that TLR-2/MyD88 signaling can under appropriate conditions contribute to *Mtb* induced Irg1 expression raises several interesting questions concerning its interplay at the cellular level with the major STING driven Type I IFN pathway described above. TLR-2 signalling under certain circumstances can trigger Type I IFN expression (Perkins and Vogel, 2015) and thus could potentially contribute to Irg1 expression by augmenting this response. Nevertheless, in our experiments TLR-2 deficient mice mounted normal IFN-β responses, thus ruling out the latter mechanism. Interestingly as discussed above, inhibition of *Mtb* phagocytosis almost completely eliminated Irg1 expression arguing that both the TLR-2 and STING dependent pathways depend on this process. Conversely, TLR-2 deficiency failed to inhibit phagocytic uptake of *Mtb* as has been described for other pathogens (Doyle et al., 2004; Letiembre et al., 2005; Shen et al., 2010) arguing against this alternative role for the receptor in Irg1 induction. TLR-2 has been shown to activate both a cytoplasmic and an endosome-dependent signaling pathway (Dietrich et al., 2010; Stack et al., 2014; Musilova et al., 2019) and based on the near total suppression of Irg1 expression resulting from inhibition of phagocytosis or endosomal acidification we speculate that TLR-2 stimulation of the gene is triggered primarily from the latter cellular site. This hypothesis is consistent with the findings of a recent study (Hinman et al., 2021) that employed Mtb mutants to demonstrate distinct early and late components of the macrophage transcriptional response to Mtb and showed that the late component requires endosomal uptake and phagosome acidification. Importantly, they identify the Irg1 gene as present in this late gene cluster that includes a major set of TLR2 induced transcripts. In contrast and in agreement with our findings, the authors of this study found that the transcript encoding TNF-α belongs to the earlier gene cluster and as such its expression is totally independent of endosomal uptake and phagosome acidification.

At present, it is unclear whether TLR-2 signaling contributes to Irg1 expression through a totally independent pathway or acts by helping prime the Type I IFN driven Irg1 response analogous to what may occur when macrophages treated with LPS are exposed to IFNs (Mills et al., 2018). Since TLR-2 signalling is not required for *Mtb* induced IFN-β expression, such a priming effect would likely take place downstream of that cytokine induction step and possibly at the level of the *Irg1* promoter itself.

Interestingly, in our experiments simultaneously measuring Irg1 reporter induction and the presence of fluorochrome expressing intracellular *Mtb* in macrophage cultures we detected Irg1 expression in both infected and uninfected bystander cells with a greater response in the former population. The stimulus for the infection independent induction of Irg1 observed in this system is presently unknown. One possibility is that it results from signals delivered by soluble bacterial components acting in combination with IFN-β and perhaps other cytokines (e.g. TNF-α) released by infected macrophages into the culture (Degrandi et al., 2009). The finding that *Mtb* triggers Irg1 expression in both cell intrinsic as well as extrinsic fashion is consistent with the potent induction of the enzyme as well as its broad effects in infected hosts (Nair et al., 2018). Indeed, in addition to triggering the innate Type I IFN driven Irg1 response studied here, in later infection *Mtb* through its induction of a strong Th1 dependent response can generate substantial levels of IFN-γ, a second major cytokine stimulus of Irg1, that should help sustain high level production of the enzyme beyond the acute phase of infection.

## Data Availability Statement

The original contributions presented in the study are included in the article/**Supplementary Material**. Further inquiries can be directed to the corresponding authors.

## Ethics Statement

All studies were conducted in accordance with protocol LPD-99E approved by the NIAID Animal Care and Use Committee.

## Author Contributions

CB and AS designed and planned all the experiments. CB, LF, EA, LM, KM, AC, SN, and DC performed the experiments. MS, MM, DM, JW, RC, DC, MD’I, and AS contributed with reagents/materials/mice/equipment/analysis tools. CB, EA, DC, and AS analyzed the data and interpreted the results. CB and AS wrote the manuscript. All authors contributed to the article and approved the submitted version.

## Funding

This study was supported by the Intramural Research Program of the NIAID, NIH (NIAID authors) and of the NCI, NIH (NCI authors) and by São Paulo Research Foundation (FAPESP-Brazil) grants: 2017/09110-4 and 2020/09043-8 (CB). 2019/08445-8 and 2019/25770-0 (DC), 2020/10356-0 (AC) and 2015/20432-8 (MD’I).

## Conflict of Interest

The authors declare that the research was conducted in the absence of any commercial or financial relationships that could be construed as a potential conflict of interest.

## Publisher’s Note

All claims expressed in this article are solely those of the authors and do not necessarily represent those of their affiliated organizations, or those of the publisher, the editors and the reviewers. Any product that may be evaluated in this article, or claim that may be made by its manufacturer, is not guaranteed or endorsed by the publisher.

## Supplementary Material

Supplementary Figure 1 *Mtb* induces *Irg1* mRNA expression *in vivo* in mouse lung and *in vitro* in murine and human macrophages. **(A)** BL/6 mice were infected with H37Rv strain (~100 c.f.u.) and the kinetics of Irg1 mRNA induction in the lungs was determined by real-time PCR. **(B)** *Irg1* mRNA expression in human monocyte-derived macrophages at 6 h post H37Rv Mtb infection (MOI 1:1). **(C)** Kinetics of *Irg1* mRNA expression was evaluated in murine macrophages upon Mtb infection (MOI 1:1). **(D**, **E)** Kinetics of *Tnfa* and *Il12a* mRNA levels as assessed in cultures described in **(C)**. **(F)** Irg1 expression induced by Mtb at different MOI. Statistically significant differences are indicated as follows: **p<0.01 and ***p<0.001). Representative results of two separate experiments performed.

Supplementary Figure 2 Phagocytosis inhibition does not reduce macrophage viability and bacterial opsonization accelerates uptake. **(A)** Cell viability of BMDMs treated with phagocytosis inhibitors or maintained in low temperature (4°C) for 7 h (equivalent to 1h of pretreatment plus 6h post-infection) measured by flow cytometry using live/dead staining. **(B)** H37Rv-RFP Mtb was opsonized with fresh or heated-inactivated normal mouse sera as described in and bacterial uptake was evaluated by flow cytometry at 1 h and 3 h p.i. Statistically significant differences are indicated as follows: **p<0.01 and ***p<0.001). Representative data from two separate experiments performed.
