# Supplementary figures and images for: Mycobacterium tuberculosis Induces Irg1 in Murine Macrophages by a Pathway Involving Both TLR-2 and STING/IFNAR Signaling and Requiring Bacterial Phagocytosis
Source: Front Cell Infect Microbiol. 2022 May 2;12:862582. doi: 10.3389/fcimb.2022.862582 (PMC9109611; doi:10.3389/fcimb.2022.862582)

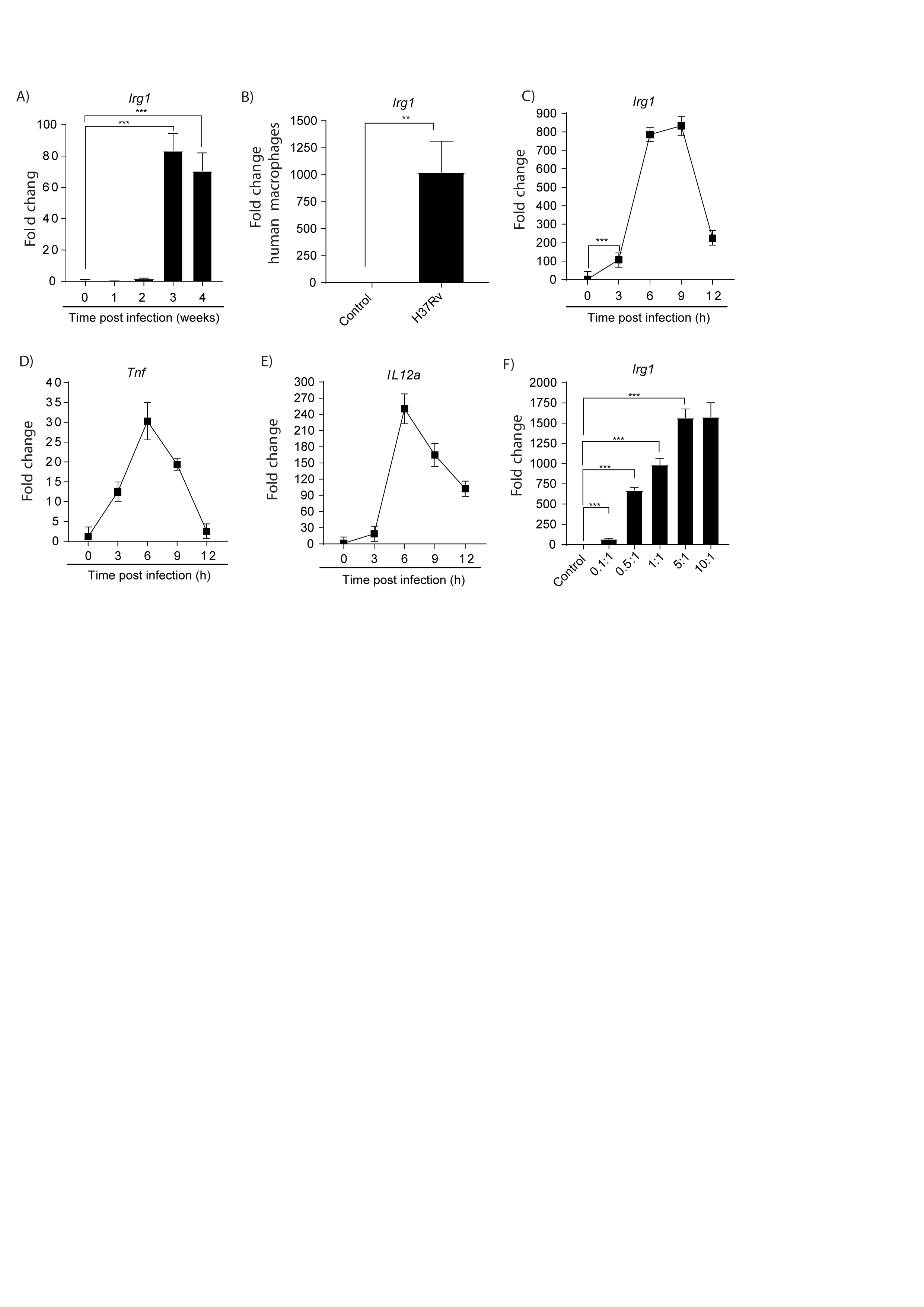

Supplement: Supplementary Figure 1 — Mtb induces Irg1 mRNA expression in vivo in mouse lung and in vitro in murine and human macrophages. (A) BL/6 mice were infected with H37Rv strain (~100 c.f.u.) and the kinetics of Irg1 mRNA induction in the lungs was determined by real-time PCR. (B) Irg1 mRNA expression in human monocyte-derived macrophages at 6 h post H37Rv Mtb infection (MOI 1:1). (C) Kinetics of Irg1 mRNA expression was evaluated in murine macrophages upon Mtb infection (MOI 1:1). (D, E) Kinetics of Tnfa and Il12a mRNA levels as assessed in cultures described in (C). (F) Irg1 expression induced by Mtb at different MOI. Statistically significant differences are indicated as follows: **p<0.01 and ***p<0.001). Representative results of two separate experiments performed. [file Image_1.tif]

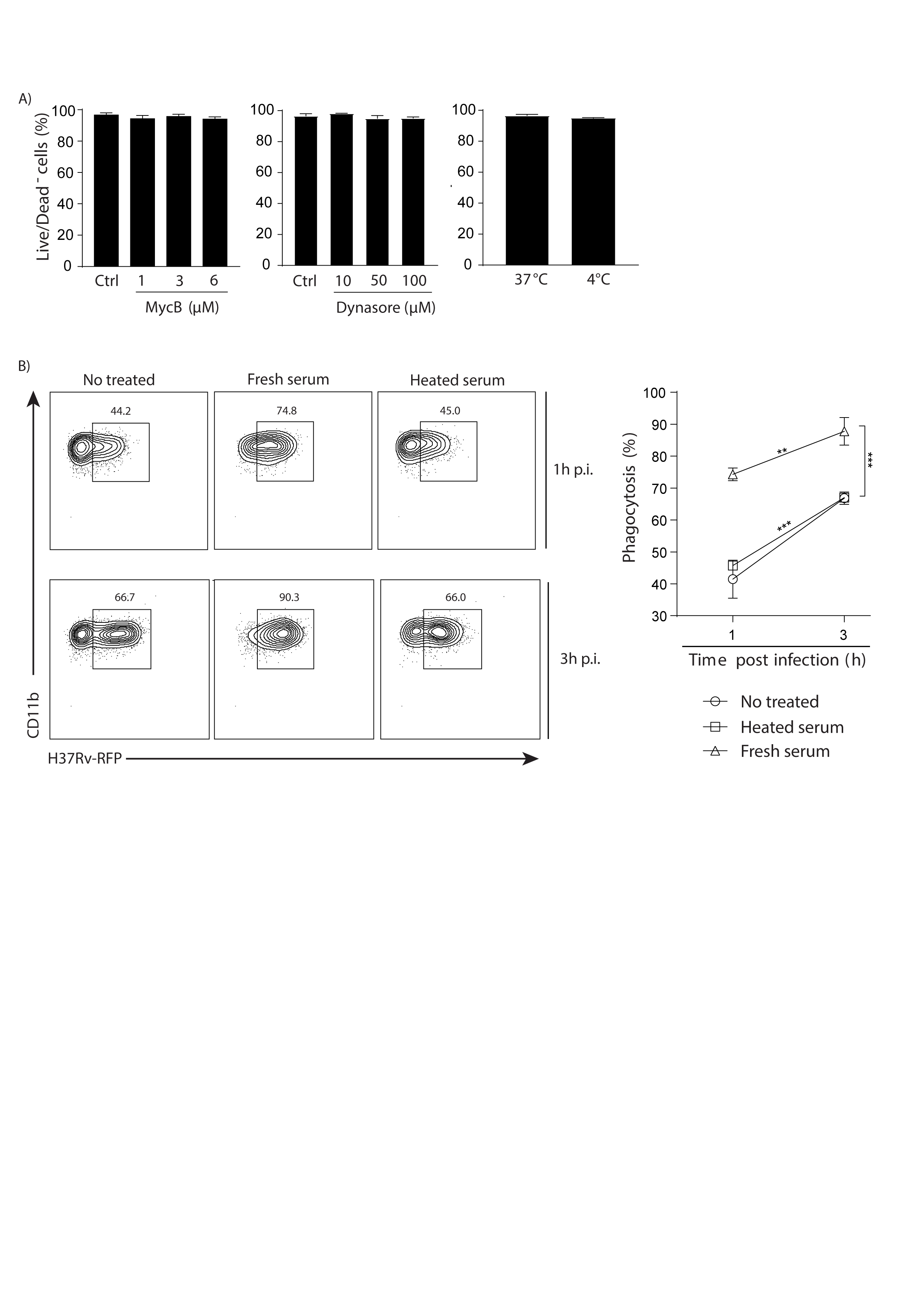

Supplement: Supplementary Figure 2 — Phagocytosis inhibition does not reduce macrophage viability and bacterial opsonization accelerates uptake. (A) Cell viability of BMDMs treated with phagocytosis inhibitors or maintained in low temperature (4°C) for 7 h (equivalent to 1h of pretreatment plus 6h post-infection) measured by flow cytometry using live/dead staining. (B) H37Rv-RFP Mtb was opsonized with fresh or heated-inactivated normal mouse sera as described in and bacterial uptake was evaluated by flow cytometry at 1 h and 3 h p.i. Statistically significant differences are indicated as follows: **p<0.01 and ***p<0.001). Representative data from two separate experiments performed. [file Image_2.tif]
